# Comment on the “Evaluation of sensory loss in the feet and associated factors in ambulatory patients with diabetes: a cross-sectional study”

**DOI:** 10.20945/2359-4292-2026-0004

**Published:** 2026-01-29

**Authors:** Han Gao

**Affiliations:** 1 Department of Nephrology, First Teaching Hospital of Tianjin University of Traditional Chinese Medicine, National Clinical Research Center for Chinese Medicine Acupuncture and Moxibustion, Tianjin, China; 2 Graduate School, Tianjin University of Traditional Chinese Medicine, Tianjin, China

Dear editor,

I read with great interest the article by Barreto and cols., titled “Evaluation of sensory loss in the feet and associated factors in ambulatory patients with diabetes: a cross-sectional study” ^(1)^. The risk of sensory loss in patients with diabetes has long been underestimated. By focusing on this condition, the authors have identified a breakthrough point for clinical management while offering patients an opportunity for early diagnosis and treatment.

The study found that approximately 20% of outpatient patients with diabetes exhibit protective sensory loss, with factors such as prolonged disease duration, target organ damage, and weight gain significantly associated with this condition — demonstrating substantial clinical and practical significance. The authors also note the unexpectedly low rate of foot screening (53% never screened), reflecting a significant gap in the prioritization of diabetic foot detection, treatment, and care — even within specialized outpatient settings.

However, I wish to raise several points for consideration:

## 1. GENERALIZABILITY AND SELECTION BIAS

Single-center sampling has limitations. Methodologically, excluding patients with prior ulcers or amputations is reasonable but may underestimate sensory loss in the broader diabetic population. Future multicenter studies encompassing the full spectrum of diabetic foot disease would provide a more comprehensive picture.

## 2. ROLE OF GLYCEMIC CONTROL

Surprisingly, despite the clear association between glycated hemoglobin (HbA1c) and diabetic neuropathy, this study did not include it as a variable. Future analyses incorporating HbA1c metrics could deepen our understanding of modifiable risk factors and guide more precise interventions.

## 3. COGNITIVE AND RECALL BIAS

The finding that 53% of patients never underwent sensory testing is concerning. However, it remains unclear whether this reflects genuine screening gaps or merely patient recall bias. Objective verification through medical records or healthcare provider reports could help distinguish between these possibilities. Healthcare providers should place greater emphasis on sensory testing. Beyond treatment approaches, cultivating awareness among both patients and physicians is equally crucial.

## 4. CLINICAL IMPLICATIONS OF “ANCHORING BIAS”

The authors’ identification of anchoring bias as a contributing factor to low screening rates among target organ damage patients offers thought-provoking insights. This highlights systemic issues in chronic disease management. I concur that integrating structured foot examination protocols — such as mandatory checklists or decision support tools — into electronic health records can effectively mitigate such biases and ensure equitable access to preventive care.

In summary, this study makes a significant contribution to diabetic foot prevention by identifying key risk factors and revealing major gaps in screening practices. It underscores the urgency of implementing systematic, protocol–driven foot care in diabetes management, particularly for high–risk populations. I anticipate this work will inspire further research and policy initiatives to enhance the early detection and prevention of diabetic neuropathy.

## Data Availability

datasets related to this article will be available upon request to the corresponding author.
